# An Economic Evaluation of TENS in Addition to Usual Primary Care Management for the Treatment of Tennis Elbow: Results from the TATE Randomized Controlled Trial

**DOI:** 10.1371/journal.pone.0135460

**Published:** 2015-08-28

**Authors:** Martyn Lewis, Linda S. Chesterton, Julius Sim, Christian D. Mallen, Elaine M. Hay, Daniëlle A. van der Windt

**Affiliations:** Arthritis Research UK Primary Care Centre, Institute of Primary Care and Health Sciences, Keele University, Keele, Staffordshire, United Kingdom; London School of Hygiene and Tropical Medicine, UNITED KINGDOM

## Abstract

**Background:**

The TATE trial was a multicentre pragmatic randomized controlled trial of supplementing primary care management (PCM)–consisting of a GP consultation followed by information and advice on exercises–with transcutaneous electrical nerve stimulation (TENS), to reduce pain intensity in patients with tennis elbow. This paper reports the health economic evaluation.

**Methods and Findings:**

Adults with new diagnosis of tennis elbow were recruited from 38 general practices in the UK, and randomly allocated to PCM (n = 120) or PCM plus TENS (n = 121). Outcomes included reduction in pain intensity and quality-adjusted-life-years (QALYs) based on the EQ5D and SF6D. Two economic perspectives were evaluated: (i) healthcare–inclusive of NHS and private health costs for the tennis elbow; (ii) societal–healthcare costs plus productivity losses through work absenteeism. Mean outcome and cost differences between the groups were evaluated using a multiple imputed dataset as the base case evaluation, with uncertainty represented in cost-effectiveness planes and through probabilistic cost-effectiveness acceptability curves). Incremental healthcare cost was £33 (95%CI -40, 106) and societal cost £65 (95%CI -307, 176) for PCM plus TENS. Mean differences in outcome were: 0.11 (95%CI -0.13, 0.35) for change in pain (0–10 pain scale); -0.015 (95%CI -0.058, 0.029) for QALY_EQ5D_; 0.007 (95%CI -0.022, 0.035) for QALY_SF6D_ (higher score differences denote greater benefit for PCM plus TENS). The ICER (incremental cost effectiveness ratio) for the main evaluation of mean difference in societal cost (£) relative to mean difference in pain outcome was -582 (95%CI -8666, 8113). However, incremental ICERs show differences in cost–effectiveness of additional TENS, according to the outcome being evaluated.

**Conclusion:**

Our findings do not provide evidence for or against the cost-effectiveness of TENS as an adjunct to primary care management of tennis elbow.

## Background

Tennis elbow is a common and painful condition and has considerable impact on daily activities, including work, with up to 30% of patients reporting time off work amounting to an average 4–5 absent work days [1,2]. The annual incidence is 1–3% in the general population [1,3,4]. Pain does not usually persist for longer than 12 months, and over 80% of patients would be expected to be better (complete or partial recovery) by 12 months [5]. The condition is predominantly managed in primary care with common interventions being rest and analgesics, non-steroidal anti-inflammatory drugs (through topical and oral administration), corticosteroid injections, and exercise. Non-steroidal anti-inflammatory drugs may have a small beneficial effect [5]; however, the risk of adverse effects may limit their usefulness in the management of pain, especially in older people. Corticosteroid injections offer significant short-term pain relief, though there are concerns over longer-term benefit and recurrence [4,6,7]. Physiotherapy-led exercises and mobilizations have also been shown to have some short-term benefit [6,8], but may be more costly. Only two cost-effectiveness evaluations are known to have been published for tennis elbow. Korthals-de-Bos and colleagues [9] found economic support for a wait-and-see approach with advice and medication, with this being more effective over the long-term than corticosteroid injections and more cost-effective than physiotherapy. Struijs and colleagues [10] found physiotherapy to be more cost-effective than the use of braces. As there is no obvious intervention of choice for the condition, there is a continued need to evaluate those interventions that may be acceptable to patients and provide safe, effective and cost-effective pain relief.

Transcutaneous electrical nerve stimulation (TENS) has been proposed as an inexpensive, safe, non-pharmacological analgesic that could provide clinical and cost-effective pain relief [11]. Currently, TENS is advocated as an adjunct to other treatments for musculoskeletal pain and promotes self-management [12]. By relieving pain and increasing self-management, the TENS intervention would likely give improved quality-of-life, and given the likely low cost of the intervention, it was anticipated that the addition of TENS would be cost-effective. Until recently, there was no information on the effectiveness of TENS in tennis elbow in a primary care population. This trial investigated the clinical and cost-effectiveness of supplementing primary care management (PCM)–consisting of a GP consultation followed by information and advice on exercises–with TENS to reduce pain intensity in patients with tennis elbow. The primary and secondary clinical outcomes of the study have been published [13]; there were no significant differences between TENS plus PCM and PCM alone for pain relief at 6 weeks, 6 or 12 months. Equally, there were no statistically significant differences between groups for secondary outcomes, which included: self-reported global change in elbow problems; limitation in function; work absence; general health; and health beliefs and perceptions. Despite this lack of difference in health outcomes, policy makers still need to consider other issues, such as costs alongside outcomes; health economic evaluation focuses on decision analysis around differences in cost and health outcome through probabilistic decision modelling techniques.

In this paper we report on the economic evaluation.

## Methods

### Study design

This was a multi-centre pragmatic two-armed randomized clinical trial that recruited adults consulting with tennis elbow from 38 general practices in the West Midlands region of the UK. Full details of the trial design, rationale, identification and inclusion/exclusion of participants, interventions, and flowchart of participants through the trial have been presented elsewhere [13,14]. In short, 241 participants were recruited to the study and randomized to receive either primary care management alone (PCM), consisting of a GP consultation followed by information and advice on exercises, or PCM plus TENS to be used once per day for 45 minutes over 6 weeks (or until symptom resolution) for pain relief. This was a pragmatic study, and although necessary concealment of the randomization sequence was performed, thereby blinding researchers and assessors to treatment allocation and assessment data, it was not possible to blind the patients and practitioners due to the nature of the proposed intervention. Postal questionnaires were used to collect outcome and health resource use data at 6-week, 6-month and 12-month follow-ups, and reminder letters were sent at two weeks and four weeks after the follow up point to non-responders. Minimal data collection of the primary outcome measure was attempted by telephone if participants still did not respond.

Ethical approval was obtained from the South Staffordshire Local Research Ethics Committee in May 2009 (reference No 09/H1203/31). All participants gave written informed consent.

### Economic perspectives

Two economic perspectives were considered: (i) a healthcare perspective, which evaluates health outcomes in relation to costs of health care resource use in primary and secondary care, and (ii) a societal perspective (primary perspective), which evaluates health outcomes in relation to a broader spread of costs that includes costs associated with lost workplace productivity due to absenteeism in addition to healthcare costs.

### Outcomes

The primary outcome of the study was reduction in elbow pain intensity from baseline. The pain intensity question at the time of assessment measured the level of elbow pain over the previous 24 hours scored on a 0–10 numerical rating scale: 0 denoting ‘no pain’ and 10 denoting ‘worst pain imaginable’. An overall 1-year pain reduction score for each individual was calculated from area-under-the-curve calculation of pain change scores by summing average values between adjacent timepoints weighted according to the width of time intervals [15]; this reflects a weighted average pain reduction score across the 1-year duration of follow-up, assuming linear interpolation between assessment timepoints. Hence, a higher overall pain reduction score indicates better health status (larger improvement) in respect of elbow pain.

The EuroQoL EQ5D-3L and Short Form 6D (SF6D) were used to measure preference-based health-related values at baseline, 6 weeks, 6 months and 12 months [16–20]. Pain and functional impairment are two important aspects of health related quality-of-life, and as such whatever influences pain (and function) is also likely to affect a person’s quality-of-life, and vice-versa. Both the EQ5D and SF6D include dimensions that embrace physical and psychosocial elements, and are valid for measurement in pain studies [21]. As these are commonly used ‘generic’ measures they allow for comparison across other health areas on the basis of the quality-adjusted life year (QALY) values, which are derived from the EQ5D and SF6D scores. QALYs were calculated from the EQ5D-3L (and separately, SF6D) health utility scores at each of the assessment timepoints using area-under-the curve methods, assuming linear interpolation between consecutive timepoints, derived by weighted average summation of values (as for 1-year pain reduction scoring described above) [15]. The ceiling QALY is 1, which is equivalent to the year's follow-up spent in full health. The minimum possible QALY values differ depending on the utility measure used to derive them: -0.59 for the EQ5D-based QALY and 0.34 for the SF6D-based QALY (denoted here as QALY_EQ5D_ and QALY_SF6D_, respectively). For the EQ5D, utility scores were derived using the ‘UK: York A1 tariff’; preference data for this tariff were elicited from a representative sample of the UK adult population, providing 243 permutations of index health state values ranging between -0.594 (denoting a state worse than death) and 1.000 (perfect health) [16]. The SF6D was derived from the Short Form 12 (SF12) [16,20]; the scoring algorithm was based on estimates from a general UK population and includes 7,500 permutations of health states ranging from 0.345 to 1.000 (though not inclusive of zero, the scoring range is still interpreted on a scale where 1 indicates full health and 0 represents a health state equivalent to death).

### Healthcare resource use and unit costs

The 6-week questionnaire collected data on resource use relating to the tennis elbow disorder between baseline and 6-week follow-up, the 6-month questionnaire collected data on resource use for the period between 6 weeks and 6 months follow-up, and the 12-month questionnaire asked about resource use related to tennis elbow between the 6-month and 12-month questionnaires. Collected resource use questions covered a range of healthcare services use: primary health care consultations (general practitioners and practice nurses–whether in clinic or at home), consultations with National Health Service (NHS) secondary care health professionals (e.g. hospital consultants, physiotherapists, radiographer, acupuncturist, osteopath, chiropractor, other…), private health care provider contacts, hospital-based investigative procedures and diagnostic tests (e.g. x-rays, scans, blood tests), prescribed medication or aids (such as elbow braces), patient-borne out-of-pocket expenditure on medication, aids and appliances, and length of any inpatient hospital stays. A distinction was drawn between UK NHS and private care provision. The publicly funded health care systems in the United Kingdom are referred to as the NHS with monetary provision sourced through the UK government; private health care costs cover a range of out-of-pocket expenditure. Unit resource costs relating to private health care visits were set at the same level as those for the corresponding NHS visits. The focus on health care resource use was specific to the patients’ tennis elbow disorder. Health resource unit costs for the economic evaluation are based on UK national average tariffs for 2011 (shown in Table 1). The 12-month questionnaire ascertaining healthcare costs and time off work (absenteeism) data is provided in S1 Appendix.

**Table 1:** Unit costs for healthcare resources.

Health care resource	Unit Cost (£)	Source	Details
***Community-based health care staff***			
General Practitioner (clinic)	£36 per consult	Curtis;[22] Section 10.8b	Average 11.7-minute consultation
General Practitioner (home)	£121 per consult	Curtis;[22] Section 10.8b	Average 23.4-minute visit
Practice nurse (clinic)	£13 per consult	Curtis;[22] Section 10.6	Average 15.5-minute consultation (£51 per hour face-to-face consult)
Practice nurse (home)	£44 per consult	Curtis;[22] Section 10.6	Based on same home: clinic cost magnification as for GPs
***Aids/appliances***			
Elbow brace	£15	Physio-med website[23]	http://www.physio-med.com/
***Hospital-based health care staff***			
Specialist (A&E)*	£32.75 per consult	Curtis;[22] Section 15.4	Estimated £131 per hour (Associate specialist) consultation. Consult cost based on assumed 15-minute consultation time.
Specialist (Outpatients)*	£40.50 per consult	Curtis;[22] Section 15.5	Estimated £162 per hour consultation. Consult cost based on assumed 15-minute consultation time.
Radiographer*	£18.50 per consult	Curtis;[22] Section 13.5	Estimated £37 per hour consultation. Consult cost based on assumed 30-minute consultation time.
Therapist and allied health practitioners*	£17.50 per consult	Curtis;[22] Section 13.1	Estimated £35 per hour consultation. Consult cost based on assumed 30-minute consultation time.
Inpatient stay	£1955	Department of Health;[24] Sheet TPCTEI	Based on weighted average figures (of 3.2 days) for HD23B/C
***Investigations***			
Plain X-ray /ultrasound	£53	Department of Health;[24] Sheet TPCT_DIAGIM_OP	Based on code RA23Z
MRI scan	£183	Department of Health [24]; Sheet TPCT_DIAGIM_OP	Based on code RA02Z
Blood test	£20	Improvement.nhs website[25]	
***Medication***	-	BNF 61[26]	Dependent on type of medication

* Unit resource costs relating to private visits to health care professionals were costed using the same rates as those for the corresponding NHS visits

### Work-absenteeism costs

To assess societal costs, inclusive of costs related to health care resource use and cost associated with work absenteeism, we collected information on patients’ work status at baseline and elbow-pain related time (days) off work at 6-weeks follow-up (for the period between baseline and 6 weeks), 6-month follow-up (for the period between 6 weeks and 6 months) and 12-month follow-up (for the period between 6 months and 12 months). For those in paid employment, the baseline questionnaire requested further details on job title, what the firm/organization makes or does, and the role of the individual within the organization. Information was sought on whether this work was typically full- or part-time. This job description information was used to code participants’ employment according to the UK Standard Occupational Classification [27]. An individual’s expected daily wage was derived using the SOC code and information for stratifying sub-samples according to gender and full-/part-time work status, and was based on national average valuations from an Office of National Statistics annual earnings survey [28,29]. Productivity costs were derived according to the human capital approach and calculated as the product of the total number of days off work (during the full 12 months of follow-up) and extracted daily wage. Non-workers, by default, did not have absenteeism cost.

### Statistical evaluation methods

The main analysis was by intention-to-treat (ITT), analysing all patients as randomized, and thereby including all patients in the group to which they were randomly assigned and imputing all missing outcome and cost data [30]. Imputation of missing cost data was in two phases. First, a number of missing frequency data for number of consultations and over-the-counter cost data were imputed using mean substitution based on the observed mean values for the respective resource category, stratified by study group. At this level, there was partially completed information; so, for example, consultation with a GP was indicated but not the frequency of consultation, and the OTC product was named but the cost of the product was not specified. Upon completion of phase 1 imputation all available and imputed resource and individual item cost data were pooled to derive aggregated cost data for all study participants. Second phase imputation involved imputation at the level of missing aggregate cost data and health outcome data, using the multiple imputation approach to impute 20 separate datasets with full data; this number of imputations was used as it is recommended that it should reasonably reflect the total participant dropout rate of the study [31]. The multiple imputation algorithm used chained equations with predictive mean matching to align missing values to the nearest best-fitting values in the dataset; this method overcomes the problem of skewness which is particularly encountered in relation to EQ5D and cost distributions. Coefficients are then pooled across the multiple imputed datasets to obtain single estimates of the corresponding population parameters [32]. The multiple imputation algorithm included all primary and secondary outcomes measured at all assessment time points (baseline, 6 weeks, 6 months and 12 months) and also aggregated costs covering the period of baseline to 6 weeks, 6 weeks to 6 months, and 6 months to 12 months for each of the following: primary care consultations; secondary care contacts; medication costs; investigation costs (all these being distinct variables according to NHS- or Private- resource classification), and cost related to lost work days. Predictor variables included baseline variables with full data including treatment group, age, gender, and baseline variables relating to outcome measures.

Though the main analysis focused on ITT evaluation of all participants, a sensitivity analysis was carried out to test the robustness of these findings by including data for all patients who completed at least partial cost data (this being based on available data following phase 1 imputation of costs), and complete-case data for health outcomes. In addition, an analysis was carried out to evaluate incremental effects and costs for the subset of 71 patients (42 in the PCM plus TENS group, 29 in the PCM group) who were treated strictly according to protocol (based on the imputed dataset).

Differences in mean outcomes and mean costs were compared between the two study groups: the PCM group was used as the reference group in the analyses. All cost and health outcome differences were derived through linear regression modelling with adjustment for the following baseline covariates: age, gender, pain score, EQ5D and SF6D.

Cost data in health care research are, typically, positively skewed. Accordingly, bias-corrected and accelerated (BCa) bootstrapping was used to derive confidence intervals for cost estimates (5000 replications) [33]. Evaluation of the joint distribution of differences in costs and health outcomes focused on the incremental cost-effectiveness ratio (ICER)–ratio of the mean difference in cost to the mean difference in effect (denoting the extra average cost per unit gain in effect for one treatment group over the other)–or otherwise dominance of one treatment over the other with respect to lower mean cost and greater mean effect. If neither treatment group is dominant, the ICER denotes an incremental cost (£) per 1-point improvement in pain reduction score, or incremental cost (£) per QALY gain in respect of EQ5D and SF6D health utility measures (referred to as the incremental cost-utility ratio [ICUR]) [34]. Analysis of uncertainty in the joint estimation of the ICER/ICUR is illustrated by cost-effectiveness/cost-utility planes (via bootstrapping with 5000 replications) [35]. Each cost-effect pair generally lies in one of four quadrants of the plane. PCM plus TENS is considered ‘dominant’ over PCM (or vice versa) if it is both more effective and less costly, i.e. if the overall mean incremental cost effectiveness estimate lies in the S-E quadrant of the plane (or, if PCM is dominant, in the N-W quadrant). When in NE or SW, the cost-effectiveness of the intervention depends on how much the decision maker is willing to pay for an additional unit of outcome. Cost effectiveness acceptability curves (CEACs) are plotted to quantify, from the bootstrap data, the probabilities of PCM plus TENS being cost effective (compared to PCM) across a range of ceiling ICER values (otherwise referred to as willingness-to-pay threshold values) [36,37].

Statistical significance was at the 5% two-tailed level. The statistical software packages used were SPSS version 20 and STATA version 13.

## Results

The baseline profile of the 241 participants, stratified by study group, has been presented elsewhere [13]. In brief, baseline characteristics were largely similar: overall mean age was 48 years, 55% were male and 43% had duration of pain exceeding 3 months.

Table 2 shows mean healthcare and societal costs for the two groups, and adjusted mean cost differences across key aggregated costs (a summary of disaggregated costs are shown in Table A in S1 File). Incremental healthcare cost was £33 for PCM plus TENS; this difference was largely due to the difference in clinic nurse consultation cost (including cost of the TENS equipment). Private healthcare costs were similar between the two study groups, and were relatively small compared to NHS costs. Mean productivity cost due to work absenteeism was £98 less for PCM plus TENS group, and resulted in an overall lower societal mean cost of £65 compared to PCM only. None of the cost differences were statistically significant.

**Table 2:** Comparison of average costs (£) per patient, by treatment group.

	PCM plus TENS(n = 121)	PCM only(n = 120)
***Healthcare costs (£)***		
Clinic nurse consultation (NHS)	52.19* (-)	13.00 (-)
Primary care consultations (NHS)	21.70 (5.14)	26.02 (6.23)
Medication/Appliances costs		
NHS	3.80 (0.90)	6.14 (1.24)
Private	11.38 (1.60)	13.77 (2.55)
Secondary care consultations (including inpatient stays)		
NHS	27.35 (18.30)	24.18 (17.42)
Private	3.41 (2.14)	0.93 (1.04)
Investigations		
NHS	11.08 (9.01)	3.35 (3.74)
Private	0.00 (0.00)	0.00 (0.00)
Total NHS cost	116.12 (26.93)	72.69 (22.02)
*Mean difference (95% CI)* †	33.59 (-35.88, 103.06)	
Total Private care cost	14.79 (3.00)	14.70 (2.71)
*Mean difference (95% CI)* †	-0.58 (-8.67, 7.51)	
Total combined NHS and Private	130.91 (28.20)	87.40 (23.35)
*Mean difference (95% CI)* †	33.01 (-40.05, 106.08)	
***Work absenteeism cost (£)*** **	149.14 (65.63)	214.58 (83.29)
*Mean difference (95% CI)* †	-98.16 (-310.14, 113.83)	
***Societal cost (£)*** ***	280.05 (80.47)	301.98 (89.49)
*Mean difference (95% CI)* †	-65.14 (-306.66, 176.38)	

Values are mean summated costs (SE) over 1 year follow-up except where mean difference in cost (95% confidence interval) is specified.

* Includes usual nurse consultation time (PCM only group) plus 5-minutes additional nurse time (additional cost, £4.19) and cost of TENS machine and pads of £35.

** 181 (75%) participants were employed at baseline (88 (73%) in the PCM plus TENS group, 93 (77%) in the PCM only group). Mean absenteeism (time off work) among workers was 1.4 (SD, 5.26) days in the PCM plus TENS group and 2.4 (SD, 8.12) days in the PCM only group. Work absenteeism costs shown in the Table are inclusive of workers and non-workers: the former being based on mean time off work and the latter being no-cost.

*** Sum of total (aggregate) healthcare cost and work absenteeism cost.

† By linear regression adjusting for baseline age, gender, pain score, EQ-5D and SF-6D.

Table 3 shows mean health outcome scores for the two groups, and adjusted mean differences in health outcomes. Mean pain reduction over follow-up was higher for PCM plus TENS than for PCM only (reflecting greater pain relief). Similarly, mean difference in QALY_SF6D_ was higher for PCM plus TENS; the converse was true for QALY_EQ5D_ where there was lower average QALY_EQ5D_ for PCM plus TENS (reflecting worse health outcome). Incremental effects were small relative to the standard deviation of corresponding baseline values, and as for costs, none of the health outcome differences were statistically significant.

**Table 3:** Comparison of average effects per patient by treatment group, and summary of ICERs relating to differences in cost and effect.

	PCM plus TENS(n = 121)	PCM only(n = 120)
***Primary clinical outcome***		
Pain change	1.771 (0.105)	1.763 (0.114)
*Mean difference (95% CI)* †	0.112 (-0.125, 0.349)	
*ICER–Healthcare costs (95% CI)*	£299.91 (-3540.25, 4107.59)	
*ICER–Societal costs (95% CI)*	-£581.61 (-8666.28, 8112.83)	
***QALYs***		
EQ-5D	0.729 (0.022)	0.767 (0.020)
*Mean difference (95% CI)* †	-0.015 (-0.058, 0.029)	
*ICER–Healthcare costs (95% CI)*	-£2200.67 (-25999.03, 22168.18)	
*ICER–Societal costs (95% CI)*	£4342.67 (-62906.66, 68824.70)	
SF-6D	0.748 (0.014)	0.765 (0.014)
*Mean difference (95% CI)* †	0.007 (-0.022, 0.035)	
*ICER–Healthcare costs (95% CI)*	£4715.71 (-37319.61, 36940.40)	
*ICER–Societal costs (95% CI)*	-£9305.71 (-104429.59, 90124.93)	

Values are mean summated values (SE) over 1 year follow-up except where mean difference (95% confidence interval) is specified.

ICER–Incremental cost effectiveness ratio = ratio of mean difference in cost to mean difference in effect (PCM plus TENS minus PCM only): denotes cost per unit increment in the ‘effect’ variable (e.g. ICER–Societal costs relative to pain (primary analysis) = -£581.61 denotes a mean cost saving of £581.61 per 1-point gain in pain outcome for the PCM plus TENS group compared to PCM only)

† By linear regression adjusting for baseline age, gender, pain score, EQ-5D and SF-6D.

**Fig 1:**
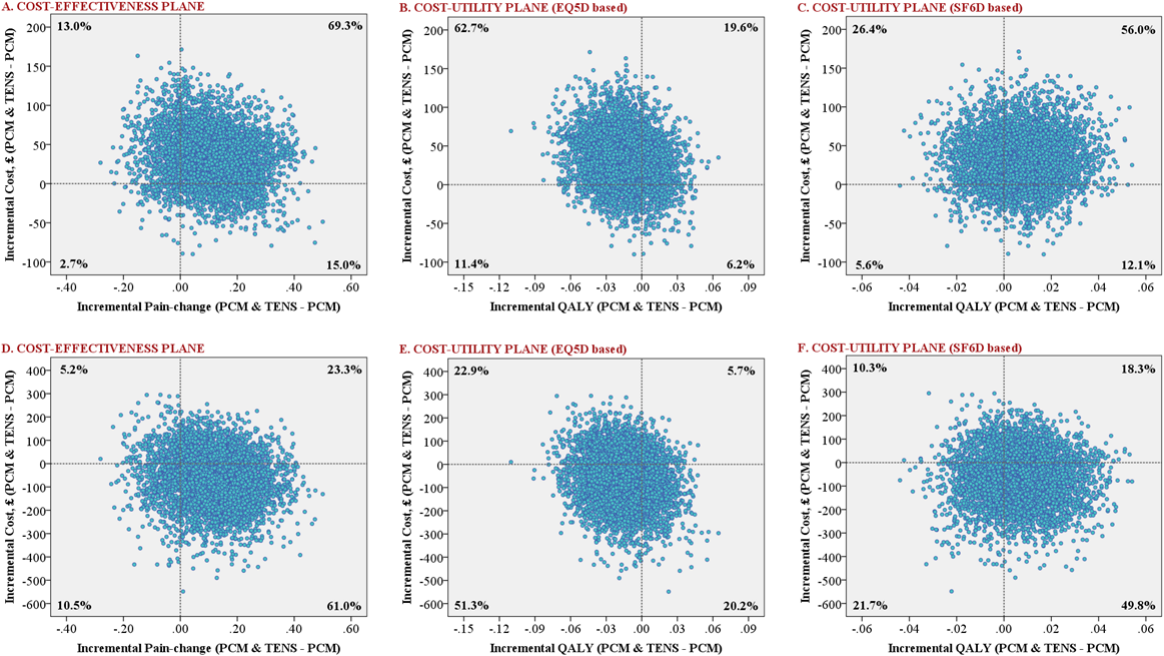
Cost-effectiveness and cost-utility planes presenting the difference in cost against the difference in effect for PCM plus TENS minus PCM only. A-C (Healthcare perspective); D-F (Societal perspective).

Cost effectiveness (utility) planes for the incremental cost effectiveness estimates are presented in Fig 1. From a healthcare perspective, dominance of PCM (over PCM plus TENS) in relation to QALY_EQ5D_ is reflected by the fact that the majority (63%) of bootstrap estimates lie in the N-W quadrant of the plane (mean cost is higher and quality of life lower for PCM plus TENS). There is no dominance in respect of pain-reduction or QALY_SF6D_. The ICER in relation to pain-reduction was £299.91 per unit (also shown in Table 3); i.e. this is the expected additional cost per 1-point improvement in pain score for the PCM plus TENS. The ICUR in relation to QALY_SF6D_ was £4715.71 per QALY; i.e. this is the expected additional cost per unit QALY gain for the PCM plus TENS. From a societal perspective, dominance of PCM plus TENS over PCM in relation to pain intensity and QALY_SF6D_ is reflected by the fact that the highest proportion (61% and 50%) of bootstrap estimates lie in the S-E quadrant. There is no dominance in respect of QALY_EQ5D_; the ICUR was £4342.67 lower per QALY loss (or conversely, this is the expected additional cost per unit QALY gain for the PCM only group).

**Fig 2:**
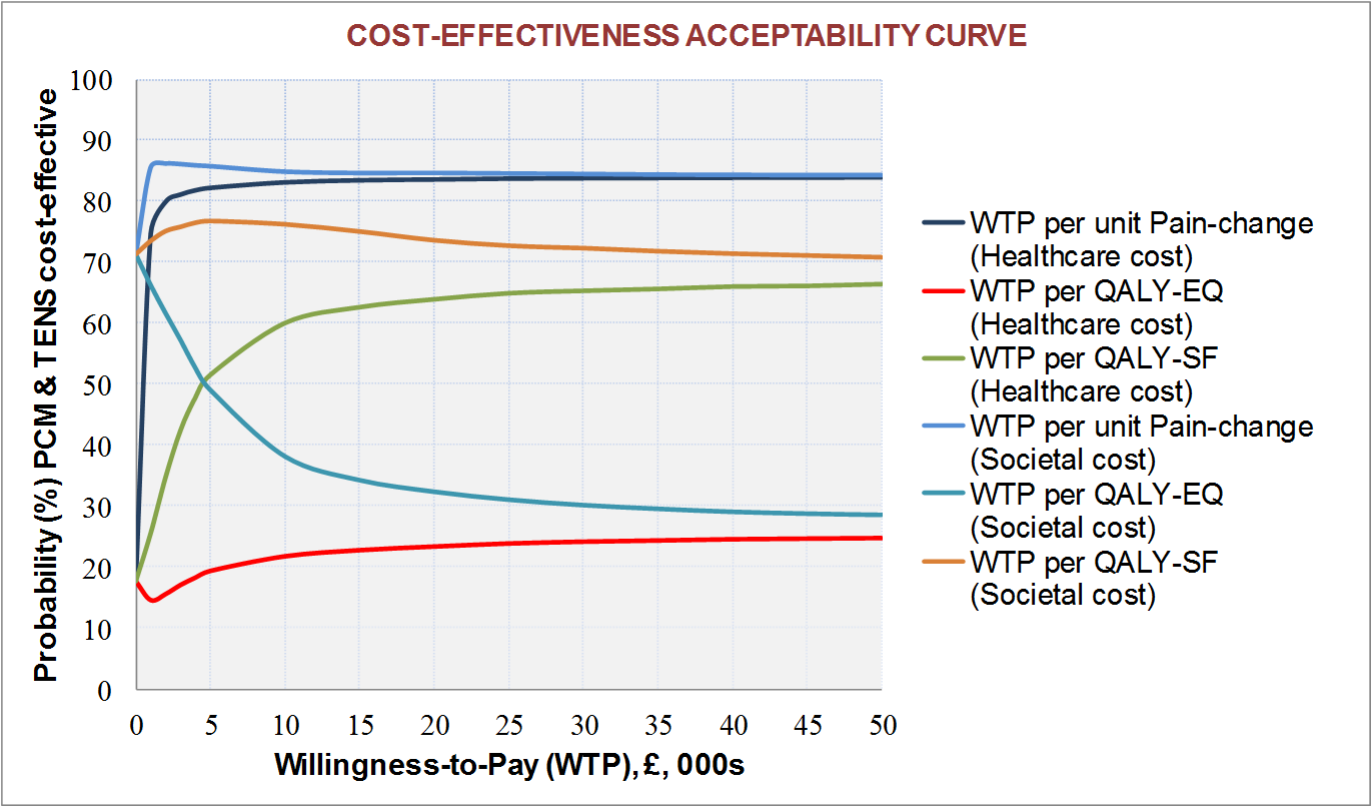
Cost-effectiveness acceptability curves showing the probability that PCM plus TENS is cost effective across different willingness-to-pay threshold values.

Cost effectiveness acceptability curves are presented in Fig 2. The graphs show that there were widely different probability values for PCM plus TENS being cost-effective compared to PCM only: ranging from 17.7% to 86.3% across willingness-to-pay thresholds up to £50,000 per unit improvement in outcome. The trend is for an increase in probability values with an increase in willingness-to-pay threshold in relation to pain-change and QALY_SF6D_; the converse of a decrease in probability values with increasing willingness-to-pay is shown for the QALY_EQ5D_.

Similar results (data shown in Tables B-C in S2 and S3 Files) were derived for the complete-case (sensitivity) analysis. For the per protocol evaluation, incremental healthcare and societal costs were respectively £56 (95%CI -81, 193) and £-174 (95%CI -580, 231), and incremental pain-change, QALY_EQ5D_ and QALY_SF6D_ were respectively 0.34 (95%CI -0.01, 0.69), -0.02 (95%CI -0.07, 0.04) and 0.00 (95%CI -0.05, 0.05). In brief, the results were similar to the base case imputed analyses with healthcare cost being higher and societal cost being lower with the addition of TENS, and overall difference in outcomes being small.

## Discussion

Our findings show that adding TENS to primary care management of tennis elbow may result in lower costs in respect of a broad societal perspective that includes health care costs and indirect costs linked to work productivity losses, but there is much uncertainty around its cost-effectiveness from both healthcare and societal health economic perspectives. The cost-effectiveness probability values were highly dependent on the outcome measure being compared: results for pain-change and QALY_SF6D_ outcomes were favourable towards additional TENS, whereas results for QALY_EQ5D_ displayed adverse effectiveness in the presence of additional TENS. As a consequence, the findings are ambiguous as to the pragmatic cost effectiveness of adding TENS to primary care management. In conjunction with the findings of the previously reported primary and secondary clinical outcomes–where there was no evidence for additional clinical benefit of TENS, albeit perhaps due in part to poor adherence to using TENS–these findings seem to indicate that adding TENS confers little advantage from a purely healthcare driven viewpoint, but that it may potentially help to lower overall societal costs.

Adding TENS to primary care management is more costly from a healthcare perspective–and the estimated cost differences over all measured primary, secondary and private health costs are due principally to the clinic cost, which includes the cost of the TENS device itself and the small additional time (5 minutes) in clinic with the nurse. It is the difference in time off work that brings about cost savings for the PCM plus TENS group; though this difference is not statistically significant and may have arisen by chance. For the 148 participants who provided complete data for time off work over the 12-month follow-up period, the mean number of days absence was 0.85 for the PCM plus TENS group compared to 1.44 days for PCM only. Though only a mean difference of 0.6 for the total study sample, it was slightly greater among the sub-group of participants with paid work at baseline (1.4 days off work versus 2.4 days, respectively). Given an estimated average weekly wage at baseline of £477 for the working sub-group, the addition of TENS may reduce average time off work by a day, resulting in a positive cost saving of around £100. The issue then is to consider why this may happen, given that clinical outcomes, including pain, function, and quality of life differed little between groups. Both groups showed considerable improvements in most clinical outcomes [13], and receiving the TENS machine may have provided patients with somewhat more confidence to return to work sooner, given their improving symptoms. Although it is the societal perspective that provides most support for TENS in this study, differences in costs and effects were small and there was considerable uncertainty in the outcome and cost differences, reflected by the wide scatter of estimates between quadrants of the cost-effectiveness planes.

At a guideline willingness-to-pay threshold of £20,000–£30,000 per QALY for healthcare expenditure (set by the National Institute for Health and Clinical Excellence (NICE), 2008 [38]), the probability of TENS (in addition to PCM) being cost-effective was about 65% in relation to QALY_SF6D_ and 24% in relation to QALY_EQ5D_. Given that health policy in the UK is based on generic measures (QALY), we cannot make strong health policy judgements about the cost-effectiveness of additional TENS as the results for the two different QALY analyses give inconsistent conclusions. Cost-effectiveness of additional TENS was clearly evident in respect of the primary outcome measure of change in pain from baseline; for example, the estimated probability that PCM plus TENS is cost-effective exceeded 80% for a cost as little as £5,000 per unit pain-change difference. From a societal perspective, the likelihood of additional TENS being cost effective was enhanced (due to more time off work in the PCM only group), but the pattern was much the same as for the healthcare perspective in that there was greater likelihood of PCM plus TENS being cost effective for two of the three outcomes (but not for QALY_EQ5D_).

One explanation for the lack of any major difference in costs and effects for the two interventions is that adherence to the TENS protocol for patients in that allocation group was low (only 30% met a priori defined adherence criteria). Poor adherence is known to be a problem in self-management of musculoskeletal disorders [39]. The recommended intention-to-treat evaluation, which was the focus of our evaluation, takes a pragmatic view to the joint comparison of costs and effects, and painted a very unclear picture regarding the cost effectiveness of TENS as an addition to primary care management for tennis elbow. Sensitivity analysis of the findings through complete case evaluation produced similar findings to the imputed datasets, thus providing support for the findings of our primary analysis. Furthermore, a per-protocol evaluation of incremental cost-effectiveness showed similar results to the base case evaluation. Aside from the limited adherence to protocol, a further limitation to this study is that not all resource costs were collected, for example, lost productivity or out-of-pocket costs relating to carers were not sought at the data collection stage. Further, the level of missing data is a concern; though a reasonable statistical approach was taken to account for missingness.

Values for the EQ5D and SF6D scales have been shown to correlate to a high degree; however, agreement of the scales is not reached at the floor- and ceiling- levels of the scales [40]. One explanation for this disparity is that the scales have a different lower- anchor; this being -0.59 for the EQ5D and 0.33 for the SF6D (although 0 is considered in both cases to be a health state equivalent to death). However, all the observed incremental effects are small, and none of the differences were statistically significant. Hence, we cannot rule out the fact that the observed differences were simply random differences; any systematic differences between the two quality-of-life scales in this context were also likely to be small.

This was an economic evaluation of a large pragmatic randomized trial evaluating a cheap and safe intervention to manage pain for tennis elbow in primary care. The main clinical finding provided no evidence to support the addition of TENS as a self-management approach to a primary care consultation consisting of advice and education. The findings of this economic study concur reasonably with the clinical findings in respect of healthcare cost-effectiveness. Thus, firm evidence is lacking and further research may be warranted to look more closely at the relationship between administration and use of TENS and work participation in patients with tennis elbow.

## Supporting Information

S1 Appendix Questionnaire for collecting information on healthcare resource use in the TATE trial at 12 months follow up. (Note: The reference interval spans from 6 months to 12 months follow up; the 6 week and 6 month questionnaires covered the intervals between baseline and 6 weeks follow up and 6 weeks and 6 months, respectively).
(DOC)

S1 File Summary of disaggregated self-report health resource use among full mailing responders to all three follow-up questionnaires. (DOCX)

S2 File Costs (£) per patient, by treatment group (complete-case analysis^#^). Values are mean costs (SE) except where mean difference in cost (95% confidence interval) is specified. ^#^ Analysis allowed for inclusion of partially completed items whereby missing data was imputed through mean substitution stratified by study group. Hence mean costs may not correspond exactly to the product of the mean number of consultations (WebTable 1) and unit cost. * Includes usual nurse consultation time (PCM only group) plus 5-minutes additional nurse time and cost of TENS machine and pads (PCM plus TENS group). ** 181 (75%) participants were employed at baseline (88 (73%) in the PCM plus TENS group, 93 (77%) in the PCM only group). Mean absenteeism (time off work) among workers was 1.4 (SD, 5.26) days in the PCM plus TENS group and 2.4 (SD, 8.12) days in the PCM only group. Work absenteeism costs shown in the Table are inclusive of workers and non-workers: the former being based on mean time off work and the latter being no-cost. *** Sum of total (aggregate) healthcare cost and work absenteeism cost. † By linear regression adjusting for baseline age, gender, pain score, EQ-5D and SF-6D. ^1–3^ Sub-sample numbers are: ^1^153 (86 in PCM plus TENS group; 67 in PCM only group); ^2^148 (80 in PCM plus TENS group; 68 in PCM only group); ^3^132 (74 in PCM plus TENS group; 58 in PCM only group).
(DOCX)

S3 File Effects by treatment group (complete-case analysis). Values are mean summated values over 1 year follow-up (area under the curve analysis) (95% confidence interval). † By linear regression adjusting for baseline age, gender, pain score, EQ-5D and SF-6D. ^1–3^ Sub-sample numbers are: ^1^162 (89 in PCM plus TENS group; 73 in PCM only group); ^2^119 (66 in PCM plus TENS group; 53 in PCM only group); ^3^103 (53 in PCM plus TENS group; 50 in PCM only group).
(DOCX)
