# Supplementary material for: An Economic Evaluation of TENS in Addition to Usual Primary Care Management for the Treatment of Tennis Elbow: Results from the TATE Randomized Controlled Trial
Source: PLoS One. 2015 Aug 28;10(8):e0135460. doi: 10.1371/journal.pone.0135460 (PMC4552676; doi:10.1371/journal.pone.0135460)
Supplement: S1 Appendix — (Note: The reference interval spans from 6 months to 12 months follow up; the 6 week and 6 month questionnaires covered the intervals between baseline and 6 weeks follow up and 6 weeks and 6 months, respectively). (DOC) [file pone.0135460.s001.doc]

**S1 Appendix. Questionnaire for collecting information on healthcare resource use in the TATE trial at 12 months follow up. (Note: The reference interval spans from 6 months to 12 months follow up; the 6 week and 6 month questionnaires covered the intervals between baseline and 6 weeks follow up and 6 weeks and 6 months, respectively).**

**This section is about treatment for this episode of tennis elbow**

The following questions are about the health professionals you have seen and treatments you have received **for** **your elbow pain** in the last **6 months** since completing your last questionnaire.

1. In the last **6 months** since completing your last questionnaire have you seen your **family doctor** or other **health care professional** who is **attached to your doctor’s practice for your tennis elbow**? Please include any home visits.

*(Please put a cross in* ***one*** box)

| Yes……. |  | *Please continue with* ***question 2*** |
| --- | --- | --- |
| No……. |  | *Please go to* ***question 3*** |

1. Please write in the table below the **number of times** you have seen each type of health professional for your **elbow problem** in the **last 6 months since completing your last questionnaire**. There are two columns: the first is to write in the number of times you have visited the GP surgery, the second is to write in the number of times the GP or health professional has visited your home.

|  | Number of times you visited the surgery | Number of times you were visited at home |
| --- | --- | --- |
| Doctor (GP) |  |  |
| Practice / district nurse |  |  |
| Other (*please specify*)  ……………………..………..... |  |  |

3. In the last 6 months since completing your last questionnaire has your doctor **prescribed you any medicines** (e.g. painkillers, anti-inflammatory drugs) **or** did you buy any medication for your current **elbow pain**?

*(Please put a cross in* ***one*** box)

| Yes……. |  | *Please continue with* ***question 4*** |
| --- | --- | --- |
| No……. |  | *Please go to* ***question 6*** |

4. Please give details of any medication you were prescribed or you have bought for your current episode of **tennis elbow** in the **last 6 months** since completing your last questionnaire.

Please put a cross to show if your doctor prescribed it, or if you brought it without a prescription. If you have used a medicine prescribed by your doctor **and** which you have also bought without prescription, please put a cross in **both** boxes.

|  | Prescribed by doctor | Bought without prescrip-tion | Number of tablets a day | For how many days? |  |
| --- | --- | --- | --- | --- | --- |
| A. Paracetamol (Panadol) |  |  |  |  |  |
| . B. Aspirin |  |  |  |  |  |
| . C. Ibuprofen tables (Nurofen, Brufen) |  |  |  |  |  |
| D. Naproxen (Naprosyn, Synflex) |  |  |  |  |  |
| E. Diclofenac (Voltarol) |  |  |  |  |  |
| F. Celecoxib (Celebrex), etoricoxib (Arcoxia), meloxicam (Mobic), or lumiracoxib (Prexige) |  |  |  |  |  |
| . G. Tramacet |  |  |  |  |  |
| . H. Co-proxamol (Distalgesic) |  |  |  |  |  |
| I. Co-dydramol |  |  |  |  |  |

|  | Prescribed by doctor | Bought without prescrip-tion | Number of tablets a day | For how many days? |  |
| --- | --- | --- | --- | --- | --- |
| J. J J. Co-codamol (e.g. Solpadol, Kapake) |  |  |  |  |  |
| K. Tramadol (e.g. Zydol, Zamadol) |  |  |  |  |  |
| . L. Dihydrocodeine (e.g. DF 118 Forte) |  |  |  |  |  |
| . M. Herbal remedies (e.g. evening primrose) |  |  |  |  |  |
| N. Other (please state)  **……………………**………………… |  |  |  |  |  |

5. We would also like to know if you have used any creams, gels, or sprays since completing your last questionnaire.

Again, please put a cross to show if your doctor prescribed it, or if you bought it without a prescription. If you have used a medicine prescribed by your doctor **and** which you have also bought without prescription, please put a cross in **both** boxes.

|  | Prescribed by doctor | | Bought without prescription | How many units (tubes, packs)? |  |
| --- | --- | --- | --- | --- | --- |
| A. Emulgel |  |  | |  |  |
| B. Feldene |  |  | |  |  |
| C. Ibuleve |  |  | |  |  |
| D. Movelat |  |  | |  |  |
| E. Powergel |  |  | |  |  |
| F. Other (please state)  ………………………… |  |  | |  |  |

1. **In the last 6 months** since completing your last questionnaire have you used an elbow brace, wrist brace, or elbow clasp?

(please put a cross in **one** box)

| Yes, prescribed by doctor…………. |  |
| --- | --- |
| Yes, bought without prescription…. |  |
| No……………………………………. |  |

1. Since completing your last questionnaire have you **been to see** any other health care professionals, either in an **outpatient** NHS service or **outpatient** **private care** facility **or** received any medical investigations (e.g. X-ray or MRI scan or blood test) for your **elbow pain**?

*(Please put a cross in one box)*

| Yes……. |  | *Please continue with* ***question 8*** |
| --- | --- | --- |
| No……. |  | *Please go to* ***question 10*** |

1. Please write in the **number of times** you have seen each of the following health care professionals for your **elbow pain**, either in the NHS or privately in the last **6 months** since completing your last questionnaire.

|  | Number of times in NHS | Number of times privately |
| --- | --- | --- |
| A. Consultant/specialist hospital doctor in **Accident & Emergency** |  |  |
| B. Consultant/specialist hospital doctor in **Outpatients** |  |  |
| C. Radiographer or imaging services |  |  |
| D. Physiotherapist |  |  |
| E. Acupuncturist |  |  |
| F. Osteopath |  |  |
| G. Chiropractor |  |  |
| H. Other (*please specify*)  …………….………............. |  |  |
|  |  |  |

9. Please write in the **number of times** you have received any of the following medical investigations, either in the NHS or privately, for your **elbow pain** in the last **6 months** since completing your last questionnaire.

|  | Number of times in NHS | Number of times privately |
| --- | --- | --- |
| A. X-ray |  |  |
| B. MRI scan |  |  |
| C. Blood test |  |  |
| D. Other (*please specify*)  ………………..…............. |  |  |

10. In the last **6 months** since completing your last questionnaire have you stayed overnight as an **inpatient** in either an **NHS service** or **private care facility (or both)** for your **elbow pain**?

| Yes……. |  | *Please continue with* ***question 11*** |
| --- | --- | --- |
| No……. |  | *Please go to* ***Next Section*** |

| 11. In the last **6 months** since completing your last questionnaire, for how many days in total were you an inpatient due to your **elbow problem**? | | | | | |
| --- | --- | --- | --- | --- | --- |
|  |  | | days |  |  |
|  |  |  | |  |
