# Supplementary material for: An Economic Evaluation of TENS in Addition to Usual Primary Care Management for the Treatment of Tennis Elbow: Results from the TATE Randomized Controlled Trial
Source: PLoS One. 2015 Aug 28;10(8):e0135460. doi: 10.1371/journal.pone.0135460 (PMC4552676; doi:10.1371/journal.pone.0135460)
Supplement: S1 File — (DOCX) [file pone.0135460.s002.docx]

Table A: Summary of disaggregated self-report health resource use among full mailing responders to all three follow-up questionnaires.

|  | PCM plus TENS (n=86) | PCM only (n=67) |
| --- | --- | --- |
| *No. of primary care consultations – Surgery (Home)* |  |  |
| GP - surgery | 32 (0) | 40 (0) |
| Nurse or other | 0 (0) | 0 (0) |
| *No. of secondary care consultations – NHS (Private)* |  |  |
| Consultant in A&E | 4 (0) | 0 (0) |
| Consultant in Outpatients | 14 (0) | 3 (0) |
| Radiographer or imaging services | 0 (0) | 2 (0) |
| Physiotherapist | 34 (0) | 5 (2) |
| Acupuncturist | 12 (3) | 0 (0) |
| Occupational health physician | 4 (0) | 0 (0) |
| Hospital inpatient stay(s) | 0 (0) | 0 (0) |
| *Investigations – NHS (Private)* |  |  |
| X-ray | 6 (0) | 2 (0) |
| MRI-scan | 4 (0) | 0 (0) |
| Blood test | 2 (0) | 0 (0) |
| *No. of responders who reported using medication – by prescription (OTC)* |  |  |
| Paracetamol | 3 (14) | 4 (14) |
| Ibuprofen tables (Nurofen, Brufen) | 4 (20) | 5 (13) |
| Ibuleve | 0 (2) | 0 (3) |
| Naproxen (Naprosyn, Synflex) | 6 (0) | 3 (0) |
| Diclofenac (Voltarol) | 2 (0) | 2 (0) |
| Co-proxamol (Distalgesic) | 2 (0) | 0 (0) |
| Co-dydramol | 2 (0) | 3 (0) |
| Co-codamol (e.g. Solpadol, Kapake) | 6 (4) | 2 (0) |
| Herbal remedies | 0 (2) | 0 (0) |
| Steroid injection | 0 (0) | 1 (1) |
| Movelat | 0 (5) | 0 (3) |
