# Supplementary material for: An Economic Evaluation of TENS in Addition to Usual Primary Care Management for the Treatment of Tennis Elbow: Results from the TATE Randomized Controlled Trial
Source: PLoS One. 2015 Aug 28;10(8):e0135460. doi: 10.1371/journal.pone.0135460 (PMC4552676; doi:10.1371/journal.pone.0135460)
Supplement: S2 File — Values are mean costs (SE) except where mean difference in cost (95% confidence interval) is specified. # Analysis allowed for inclusion of partially completed items whereby missing data was imputed through mean substitution stratified by study group. Hence mean costs may not correspond exactly to the product of the mean number of consultations (WebTable 1) and unit cost. * Includes usual nurse consultation time (PCM only group) plus 5-minutes additional nurse time and cost of TENS machine and pads (PCM plus TENS group). ** 181 (75%) participants were employed at baseline (88 (73%) in the PCM plus TENS group, 93 (77%) in the PCM only group). Mean absenteeism (time off work) among workers was 1.4 (SD, 5.26) days in the PCM plus TENS group and 2.4 (SD, 8.12) days in the PCM only group. Work absenteeism costs shown in the Table are inclusive of workers and non-workers: the former being based on mean time off work and the latter being no-cost. *** Sum of total (aggregate) healthcare cost and work absenteeism cost. † By linear regression adjusting for baseline age, gender, pain score, EQ-5D and SF-6D. 1–3 Sub-sample numbers are: 1153 (86 in PCM plus TENS group; 67 in PCM only group); 2148 (80 in PCM plus TENS group; 68 in PCM only group); 3132 (74 in PCM plus TENS group; 58 in PCM only group). (DOCX) [file pone.0135460.s003.docx]

Table B: Costs (£) per patient, by treatment group (complete-case analysis^#^).

|  | PCM plus TENS | PCM only |
| --- | --- | --- |
|  |  |  |
| ***Healthcare costs (£)***^1^ |  |  |
| Clinic nurse consultation (NHS) * | 52.19 (-) | 13.00 (-) |
| Primary care consultations (NHS) | 14.23 (3.83) | 24.33 (6.71) |
| Medication/Appliances costs |  |  |
| NHS | 2.78 (0.70) | 5.47 (1.43) |
| Private | 11.29 (1.68) | 15.57 (3.43) |
| Secondary care consultations (including inpatient stays) |  |  |
| NHS | 18.51 (14.76) | 5.00 (2.02) |
| Private | 0.92 (0.92) | 0.78 (0.78) |
| Investigations |  |  |
| NHS | 12.67 (12.44) | 1.85 (1.60) |
| Private | 0.00 (0.00) | 0.00 (0.00) |
| Total NHS healthcare cost | 100.39 (28.57) | 49.65 (10.35) |
| *Mean difference (95% CI)†* | 48.81 (-16.26, 113.88) | |
| Total Private healthcare cost | 12.20 (2.02) | 16.36 (3.48) |
| *Mean difference (95% CI)†* | -4.72 (-12.43, 2.98) | |
| Total combined NHS and Private | 112.59 (29.17) | 66.01 (12.36) |
| *Mean difference (95% CI)†* | 44.09 (-23.23, 111.40) | |
|  |  |  |
| ***Work absenteeism cost (£)*****^2^ | 66.29 (36.32) | 128.48 (69.38) |
| *Mean difference (95% CI)†* | -91.95 (-243.43, 59.54) | |
|  |  |  |
| ***Societal cost (£)******^3^ | 189.70 (68.09) | 219.31 (85.21) |
| *Mean difference (95% CI)†* | -73.00 (-288.63, 142.63) | |
|  |  |  |
