# Supplementary material for: An Economic Evaluation of TENS in Addition to Usual Primary Care Management for the Treatment of Tennis Elbow: Results from the TATE Randomized Controlled Trial
Source: PLoS One. 2015 Aug 28;10(8):e0135460. doi: 10.1371/journal.pone.0135460 (PMC4552676; doi:10.1371/journal.pone.0135460)
Supplement: S3 File — Values are mean summated values over 1 year follow-up (area under the curve analysis) (95% confidence interval). † By linear regression adjusting for baseline age, gender, pain score, EQ-5D and SF-6D. 1–3 Sub-sample numbers are: 1162 (89 in PCM plus TENS group; 73 in PCM only group); 2119 (66 in PCM plus TENS group; 53 in PCM only group); 3103 (53 in PCM plus TENS group; 50 in PCM only group). (DOCX) [file pone.0135460.s004.docx]

Table C: Effects by treatment group (complete-case analysis).

|  | PCM plus TENS | PCM only |
| --- | --- | --- |
|  |  |  |
| ***Primary clinical outcome*** |  |  |
| Pain change | 1.945 (0.115) | 1.803 (0.141) |
| *Mean difference (95% CI)†* | 0.178 (-0.070, 0.426) | |
|  |  |  |
| ***QALYs*** |  |  |
| EQ-5D | 0.735 (0.026) | 0.793 (0.022) |
| *Mean difference (95% CI)†* | -0.014 (-0.068, 0.041) | |
| SF-6D | 0.741 (0.017) | 0.771 (0.017) |
| *Mean difference (95% CI)†* | 0.004 (-0.032, 0.041) | |
|  |  |  |
